# The effects of exercise on cardiometabolic outcomes in women with polycystic ovary syndrome not taking the oral contraceptive pill: protocol for a systematic review and meta-analysis

**DOI:** 10.1186/s13643-019-1030-8

**Published:** 2019-05-16

**Authors:** Amie Woodward, Markos Klonizakis, Ian Lahart, Anouska Carter, Caroline Dalton, Mostafa Metwally, David Broom

**Affiliations:** 1 Faculty of Health and Wellbeing, Sheffield Hallam University, Collegiate Crescent, Sheffield, S10 2BP UK; 2 Institute of Sports and Human Science, University of Wolverhampton, Wolverhampton, WV1 1LY UK; 3 Faculty of Health and Wellbeing, Sheffield Hallam University, Howard Street, Sheffield, S1 1WB UK; 4 Sheffield Teaching Hospitals NHS Foundation Trust, Jessop Wing, Tree Root Walk, Sheffield, S10 2SF UK

**Keywords:** Polycystic ovary syndrome, Cardiovascular risk, Androgens, Exercise intervention, Lipid profile, Inflammation markers, Systematic review

## Abstract

**Background:**

Polycystic ovary syndrome (PCOS) is the most common endocrinopathy, affecting 4–12% of reproductive-aged women. Women with PCOS often exhibit many metabolic abnormalities that are associated with an increased cardiovascular disease (CVD) risk, independent of obesity. Exercise interventions from 12 to 24 weeks have been shown to have positive effects on blood lipid profile, ovulation and insulin resistance in women with PCOS. However, no consensus on which exercise interventions are effective (i.e. duration, type of exercise, frequency), including for different phenotypes, currently exists. The aim of this systematic review and meta-analysis is to define effective types of exercise interventions to improve cardiometabolic profile, across the range of phenotypes of PCOS.

**Methods:**

We will conduct electronic database searches, including randomised-controlled trials (RCT), quasi-RCT and clinical trials. Primary outcomes sought will be lipid profile, carotid-intima media thickness, fasting blood glucose, %HbA1c, blood pressure, waist circumference, waist-to-hip ratio, abdominal adiposity and inflammation markers. Secondary outcomes sought will be free and total testosterone, sex hormone binding globulin and insulin resistance. The Cochrane Risk Assessment Tool will be used to assess study quality. Data will be analysed in RevMan. Analysis of heterogeneity will be undertaken using the *I*^*2*^ statistic. Significant heterogeneity will be explored, and sensitivity analyses carried out as appropriate. A subgroup analysis based on androgen profile will be undertaken if data are sufficient.

**Discussion:**

A large proportion of women are affected by PCOS. It is prudent to examine how CVD risk can be mitigated in this high-risk population, and this review aims to provide evidence-driven recommendations on the types of exercise interventions that are effective for this. The review will seek to provide recommendations regarding type, frequency and duration of exercise interventions to improve cardiometabolic profile in PCOS. The subgroup analysis may be able to highlight difference in intervention effects between normo-androgenic and hyper-androgenic profile.

Limitations include heterogeneity across studies and a scarcity of clinical trials involving a PCOS control group not undertaking any intervention.

**Systematic review registration:**

PROSPERO CRD42018086117

## Background

### Introduction to polycystic ovary syndrome

Polycystic ovary syndrome (PCOS) is the most common endocrinopathy, with 4–12% of reproductive-aged women affected [1]. The typically used diagnostic criteria in the UK are the Rotterdam Criteria, which resulted from a conference sponsored by the European Society of Human Reproduction and Embryology (ESHRE) and the American Society for Reproductive Medicine (ASRM) [2]. The Rotterdam Criteria requires that women present with at least two of the three signs/symptoms to receive a diagnosis [2]. Those three criteria are clinical or biochemical hyperandrogenism, anovulation or oligomenorrhea, and polycystic ovaries, in the absence of other pathologies that can promote these symptoms [2].

Women with PCOS often exhibit many metabolic abnormalities that are associated with an increased CVD risk, independent of obesity [3]. These include insulin resistance, impaired glucose tolerance (IGT), dyslipidaemia, type 2 diabetes (T2D), hypertension, subclinical atherosclerosis, abdominal obesity, and a two to fourfold higher prevalence of metabolic syndrome compared to body mass index (BMI)-matched women [4–7]. It has been reported that the prevalence of T2D among women with PCOS is 2.6 times higher than that of the general female population [8]. This risk steadily increases with BMI and is particularly high for obese women [8]. However, it is not clear, due to a lack of long-term follow-up studies, whether this increased risk is solely attributed to obesity. It is estimated that 33% to 50% of women with PCOS are overweight or obese [9], which indicates that obesity is not the only factor influencing the prevalence or severity of PCOS [9]. Dyslipidaemia, characterised by high triglyceride (TG) and low high-density lipoprotein (HDL) concentrations, is prevalent in up to 70% of women with PCOS [5]. Consequently, women with PCOS have a 50% increased risk of cardiovascular events compared to their weight-matched counterparts [6].

Inflammatory markers that are implicated in the mediation of CVD have been reported to be increased in PCOS [10]. These range from high-sensitivity C-reactive protein [11, 12] to increased white cell count, neutrophil/lymphocyte ratio, tumour-necrosis factor-alpha (TNF-a), and interleukin-6 (IL-6) [12–15]. Moreover, various studies have reported that carotid intima-media thickness (cIMT), a marker of subclinical atherosclerosis, is higher in PCOS women compared to controls [16].

Lifestyle interventions and modifications are widely considered to be a cornerstone of PCOS treatment for cardiometabolic symptoms [2, 17]. A 2011 systematic review examining the effects of exercise interventions on PCOS found improvements in lipid profile, ovulation, and insulin sensitivity by up to 30%, independent of weight loss, within 12 weeks and across eight studies [18]. This is important for lean women with PCOS who present with insulin resistance and associated CVD risk factors, because they may still benefit from exercise despite their normal BMI. However, it does appear that positive results are maximised when exercise is concurrent with weight loss, and this appears to be achievable through longer duration interventions [18].

Various systematic reviews have been conducted to identify the effects of lifestyle interventions in PCOS, to the authors’ knowledge, there have been no recent reviews that isolate the effects of exercise and exclude studies with lifestyle interventions, with a meta-analysis conducted on cardiometabolic outcomes.

Therefore, the current systematic review and meta-analysis aim to provide data-backed recommendations on type, frequency, and duration of exercise interventions specifically aiming to improve cardiometabolic profile in women with PCOS via the following objectives:

1. Conduct an updated systematic review that will include primary studies published up to April 2018.
2. Undertake an in-depth analysis of cardiometabolic outcomes solely, without sharing the focus with fertility-related outcomes.
3. Dependent on sufficient androgen data, aim to partition results based on androgen profile, such that any difference between normo-androgenic and hyper-androgenic phenotypes will be highlighted.
4. Include only control groups containing women with PCOS undertaking no intervention or standard care, so that the effects of exercise can be isolated.

## Methods

The review will be reported in accordance with the Preferred Reporting Items for Systematic Reviews and Meta-Analysis (PRISMA) guidance. This protocol has been summarised according to the Preferred Reporting Items for Systematic review and Meta-Analysis Protocols (PRISMA-P) and has been registered in the International Prospective Register of Systematic Reviews (PROSPERO).

### Eligibility criteria

RCT, quasi-RCT and clinical trials will be screened according to population, intervention, comparison, and outcome (PICO) criteria as highlighted in Table 1:

**Table 1:** Inclusion and exclusion criteria

PICO	Inclusion criteria	Exclusion criteria
Population	Diagnosed with PCOS according to the Rotterdam Criteria 2003, National Institute of Health (NIH) 1990 criteria or Androgen Excess and Polycystic Ovary Syndrome (AE-PCOS) Society 2006 criteria. Be post-menarche and pre-menopausal. Be inactive (< 150 min/week of moderate-to-high intensity physical activity).	Receiving fertility treatment, taking metformin or OCP, or having a diagnosis of any pathology that may be promoting PCOS symptoms such as Cushing’s syndrome, congenital hyperplasia or androgen-secreting tumour.
Intervention	Any sample size. Aerobic exercise training, anaerobic exercise training, resistance training or combinations. At least 2 weeks of structured, supervised sessions. Interventions including multiple arms (such as aerobic and anaerobic exercise training, or a medication arm) will be included if it is possible to isolate the effects of the exercise intervention through a control group or placebo.	Crossover trials and interventions that are combined (such as lifestyle intervention including both exercise and diet management—where diet management refers to participants actively changing their caloric intake or the macronutrient composition of their diet in response to given targets).
Comparison	A control group of women with PCOS undertaking no interventions. No intervention is defined as not taking part in any structured exercise training and not receiving fertility treatment, metformin, oral contraceptive pill (OCP) or statins.	Healthy control group.
Outcome	Primary outcomes will be low-density lipoprotein cholesterol (LDL-C), high-density lipoprotein cholesterol (HDL-C), total cholesterol (TC), TC:HDL ratio, TG, oxidised LDL, cIMT, fasting glucose, HbA1c, blood pressure, waist circumference (WC), waist-to-hip ratio (WHR), abdominal adiposity and inflammation markers. Secondary outcomes will be total testosterone, free testosterone, sex hormone binding globulin (SHBG), fasting insulin and homeostatic model assessment for insulin resistance (HOMA-IR).	Outcome measures that have not been recorded at baseline and post intervention.

### Searches

The electronic databases as follows will be searched from inception to present: CINAHL Complete (EBSCO), Cochrane Central Register of Controlled Trials (CENTRAL) (Wiley), MEDLINE (EBSCO), Scopus (Elsevier), SPORTDiscus (EBSCO), PEDro (The University of Sydney) and PubMed (US National Library of Medicine). Clinical trials will be sought via searches of ClinicalTrials.gov and UK Clinical Trials Gateway. Only English language publications will be sought, and no publication date limitations will be applied.

The search terms will be PCOS or polycystic ovary syndrome and terms relating to exercise or physical activity interventions. These will be adapted for use in other databases.

The PubMed search strategy can be found in the Appendix.

### Data collection and analysis

#### Study selection

Results from the database searches will be imported into RefWorks and de-duplicated once the searches are complete. The title and abstract for each paper will then be exported to Microsoft Excel. Screening will be undertaken in Microsoft Excel, with one reviewer (AW) screening each result. A second reviewer (MK and DB) will screen each result in duplicate. Cohen’s kappa statistic will be used to determine inter-rater reliability.

The full-text will then be retrieved for each of the initially included studies, such that they can be examined in more detail to determine their relevancy. This will be undertaken by AW with screening undertaken in duplicate by a second reviewer (MK or DB). A reason will be provided for any studies excluded at this stage. Throughout this stage, disagreement between two reviewers will result in discussion and input from a third reviewer until a consensus is reached. Where the full-text is not available, a request will be made to the British Library.

#### Data extraction

A data extraction form will be created in Microsoft Excel which will be piloted using at least two studies. One reviewer will extract data using the form, and all data will be double-checked for consistency by a second reviewer (MK or DB).

Extracted data will include bibliographic information (such as title, journal, primary author, publication date, count and type of study arms and sample sizes), study characteristics (such as study design, count and type of study arms and sample sizes), participant characteristics (such as age and BMI and PCOS diagnostic criteria), intervention and comparison data (such as type of control group, type, duration and frequency of exercise intervention, randomisation and withdrawal) and outcome data including any relevant parameters named in the primary and secondary outcomes, taken at baseline before intervention, and post-intervention, for each arm.

In the case of any missing or unclear data, two attempts will be made to contact the corresponding author by email. If no response is received, the study will be excluded. Data will be extracted as mean and standard deviation (SD) for baseline and post-intervention values. *P* values and confidence intervals will also be extracted if available.

### Risk of bias in individual studies and heterogeneity

The Cochrane Risk Assessment Tool will be used to assess quality at the study level. The tool evaluates studies based on six criteria: (1) randomisation generation, (2) allocation concealment, (3) blinding of outcome assessors, (4) incomplete outcome data (that is, lost to follow-up), (5) selective outcome reporting and (6) other risks of bias. Because it is not possible to blind participants to their intervention allocation due to the demands of studies requiring engagement with exercise programmes, we did not include this domain in the risk assessment.

Heterogeneity of results will be assessed using the *I*^*2*^ statistic. This statistic measures the consistency of results across studies, that is, whether the variation in outcomes across studies is due to chance (homogeneity) or whether there are genuine, underlying differences between the studies (heterogeneity) [19]. This statistic has been chosen for its simplicity and applicability to meta-analyses regardless of the number of studies involved [19]. A result of over 50% will be considered significant heterogeneity. We will then choose whether a random effect or fixed effect model will be most appropriate for meta-analysis. Sensitivity analyses will be performed as appropriate.

### Data synthesis

Data that meets the inclusion criteria of outcomes measured and presented pre- and post-intervention will be quantitatively synthesised. Outcomes will be recorded in tables outlining means and standard deviation, with effect size expressed as mean difference (difference between means) with 95% confidence intervals and study weighting. The mean difference will be calculated as the difference between final (post-intervention) values rather than change scores. This is because baseline and final values may be reported for different numbers of participants due to missed visits or study withdrawals, leading to inaccurate change scores [20]. In addition, change scores are often not presented with standard deviations and imputing them may be inappropriate because of differences between studies [20]. However, in cases where there are significant differences at baseline, change scores may be used if it is appropriate to impute SD. In cases where only the change score is available, efforts will be made to contact authors to obtain final value scores. If this is not possible, change scores will be included if presented with a SD. If there is no SD, it may be imputed where appropriate.

Data will be parsed according to the review question, i.e. by type, duration and frequency of intervention. Forest plots will be generated and a *P* value of < 0.05 will be considered statistically significant.

Statistical analysis will be undertaken using RevMan 5 [21].

### Subgroup analysis

If enough androgen data is available, data will be partitioned into normo-androgenic or hyper-androgenic profiles, based on a free testosterone measure, where > 11 pmol/L indicates hyper-androgenism [22]. Hence, differences between these phenotypes will be highlighted.

To perform the subgroup analysis, free testosterone data is needed and a sufficient level of homogeneity between such studies to parse the results by androgen profile. If this is inappropriate, descriptive statistics and commentary will be provided in place of a formal meta-analysis.

If data are available, subgroup analysis will be performed on type, frequency and duration of exercise intervention.

### Confidence in findings

The Grading of Recommendations Assessment, Development and Evaluation (GRADE) will be used to grade the quality of the evidence and the strength of a finding. GRADE provides a systematic and explicit approach to making judgements about clinical and healthcare guidelines and recommendations, based on the quality of the evidence behind them. The use of a consistent and transparent approach to evaluating recommendations increases the facilitation of critical appraisal and improves communication of these judgements [23].

## Discussion

PCOS is a complex hormonal and metabolic disorder characterised by higher amounts of visceral fat, obesity, dyslipidaemia and insulin resistance. It is associated with reproductive and cardiometabolic complications. Management of PCOS is usually dependent on both the symptoms and suspected causes of PCOS. However, since PCOS is often presumed to be the result of an endocrine system feedback loop, it can be a challenge to isolate a single influence [24]. Thus, treatment modes vary, encompassing drug treatment with metformin or the oral contraceptive pill (OCP) [25], surgery [26] and alternative treatments such as acupuncture [27].

Phenotypes that present with hyperandrogenism have been shown to have a worse metabolic profile and increased risk factors than other phenotypes, despite comparable distributions of body weight [28, 29]. This may be because insulin acts as a co-gonadotrophin to stimulate ovarian androgen production, and thus hyperinsulinaemia and the severity of insulin resistance is associated with increased circulating androgen concentrations [24]. Indeed, the increased circulating androgens may then contribute to the accumulation of abdominal fat, further exacerbating insulin resistance. Increased androgens may also play a role in inflammation in PCOS, by promoting adipocyte hypertrophy and increasing mononuclear cell sensitivity to glucose, which in turn stimulates mononuclear cells to release TNF-a and IL-6 [30].

Although exercise interventions have been conducted with women with PCOS, there are no guidelines for the higher risk phenotype, or indeed any guidance on which exercise interventions are most effective for differing phenotypes, regarding duration, type of exercise, and frequency of exercise sessions. Furthermore, no reviews have attempted to highlight the difference between the normo-androgenic and hyper-androgenic phenotypes of PCOS in response to exercise interventions. Subsequently, the objective of this systematic review and meta-analysis is to define effective types of exercise interventions to improve cardiometabolic profile, across the range of phenotypes of PCOS.

Potential limitations include heterogeneity across studies due to the inclusion of exercise interventions of all types/durations/frequencies and a scarcity of clinical trials involving a control group that not only has PCOS, but also is not undertaking any other intervention; many of the exercise trials for PCOS involve a diet or ‘lifestyle’ intervention arm, whereas this review will look to isolate the effects of exercise. A further limitation is that only studies published in the English language will be included; this has the potential to introduce language or cultural bias.

## Appendix

The search was performed using PubMed syntax as demonstrated below, and amended for other databases:

1. “polycystic ovary syndrome”
2. PCOS
3. PCO*
4. polycystic ovar*
5. stein leventhal
6. (1 OR 2 OR 3 OR 4 OR 5)
7. Exercise[majr]
8. exercise therapy [majr]
9. Physical activity [majr]
10. Physical Fitness [majr]
11. Physical Endurance[majr]
12. exercis*[tw]
13. ((physical or motion) AND (fitness or therapy or therapies))[tw]
14. ((strength or resistance or circuit or enduran* or aerob* or physic* or fit*) AND train*)[tw]
15. (7 OR 8 OR 9 OR 10 OR 11 OR 12 OR 13 OR 14)
16. (6 AND 15)

((((((("polycystic ovary syndrome"[Title/Abstract]) OR PCOS[Title/Abstract]) OR PCO*[Title/Abstract]) OR polycystic ovar*[Title/Abstract]) OR stein leventhal[Title/Abstract]))) AND (((((((Exercise[majr]) OR exercise therapy [majr]) OR Physical activity [majr]) OR Physical Endurance[majr]) OR exercis*[tw]) OR (((physical or motion) AND (fitness or therapy or therapies))[tw])) OR (((strength or resistance or circuit or enduran* or aerob* or physic* or fit*) AND train*)[tw]))

## Abbreviations

AE-PCOS: Androgen Excess and Polycystic Ovary Syndrome
BMI: Body mass index
cIMT: Carotid intima-media thickness
CVD: Cardiovascular disease
GRADE: Grading of Recommendations Assessment, Development and Evaluation
HDL: High-density lipoprotein
HOMA-IR: Homeostatic model assessment of insulin resistance
IL-6: Interleukin-6
LDL: Low-density lipoprotein
NIH: National Institute of Health
OCP: Oral contraceptive pill
PCOS: Polycystic ovary syndrome
PRISMA-P: Preferred Reporting Items for Systematic Reviews and Meta-Analysis Protocols
SHBG: Sex hormone binding globulin
TC: Total cholesterol
TG: Triglycerides
TNF-a: Tumour necrosis factor-alpha
WC: Waist circumference
WHR: Waist to hip ratio
